# Charles Darwin’s Mitochondrial Disorder: Possible Neuroendocrine Involvement

**DOI:** 10.7759/cureus.20689

**Published:** 2021-12-25

**Authors:** John Hayman, Josef Finsterer

**Affiliations:** 1 Clinical Pathology, The University of Melbourne, Melbourne, AUS; 2 Neurology, Krankenanstalt Rudolfstiftung, Vienna, AUT

**Keywords:** charles darwin’s illness, somatostatin, serotonin, functional abdominal pain syndrome, idiopathic flushing, cyclic vomiting, panic disorder, neuroendocrine dysfunction, melas syndrome, mitochondrial disorder

## Abstract

Charles Darwin, the famous naturalist, suffered relapsing, debilitating illness for most of his adult life with a plethora of symptoms. The diagnosis favoured here for this illness is that of an adult-onset mitochondrial disorder due to a maternally inherited, pathological mitochondrial DNA mutation clinically manifesting as MELAS (mitochondrial encephalopathy, lactic acidosis, and stroke-like episodes) syndrome. This diagnosis accounts for Darwin’s primary symptoms; in addition, it accounts for the various unusual illnesses that afflicted his siblings and maternal (Wedgwood) ancestors.

Symptoms of Darwin’s illness may be related to dysfunction of cells with high energy requirements; this includes cells constituting the cardiac conduction system, cerebral endothelial cells, neurons, neuroepithelial cells of the vestibular apparatus, and, as proposed here, central and peripheral neuroendocrine cells.

Although Darwin’s episodes of sudden facial flushing, his nocturnal panic attacks, and his severe gastrointestinal symptoms are not readily explained, these symptoms may relate to neuroendocrine dysfunction, either an uncontrolled release of stimulatory hormone or impaired inhibitory control. It is also conceivable that the autonomic system had been involved. A study of Darwin’s illness may benefit those who suffer from similar symptoms today.

## Introduction and background

Darwin’s Illness

Joseph Dalton Hooker (1817-1911) became a close friend of Darwin (Charles Robert Darwin 1809-1882) and would stay with the family in their home near Downe in Kent, south of London, when they would have early morning discussions. In his ‘Reminiscences’ he wrote: *‘These morning interviews were followed by his taking a complete rest, for they always exhausted him, often producing a buzzing noise in the head, and sometimes what he called “stars in the eyes”, the latter too often the prelude of an attack of violent eczema in the head, during which he was hardly recognisable*’ [1].

This description by a close friend gives four of Darwin’s many symptoms related to his relapsing illness. To a one-time clinician ‘violent eczema in the head’ and ‘hardly recognisable’ suggests a carcinoid flush [2]. Darwin did not have a carcinoid tumour, the usual cause of such flushing. With a tumour he would not have lived to the age of 73, dying of an unrelated heart condition. This particular symptom may be considered an example of ‘idiopathic flushing’ (IF) [3].

Darwin’s symptoms have been described elsewhere in detail [4,5]. A list of these symptoms together with an interpretation of them is presented in Table 1. It should be noted that in addition to the transient exhaustion described by Hooker he also suffered from chronic fatigue such that he could ‘only lie on a sofa and do nothing’ [6]. He also suffered from panic attacks, waking at night feeling ‘terribly afraid’, attacks that were also associated with sweating and palpitations [7].

**Table 1:** Symptoms experienced by Darwin along with their interpretation. CVS: cyclic vomiting syndrome; WPW: Wolff-Parkinson-Wight syndrome; CFS: chronic fatigue syndrome; DSM-5: Diagnostic and Statistical Manual of Mental Disorders, Fifth Edition; ACTH: adrenocorticotropic hormone; MSH: melanocyte-stimulating hormone

Symptoms	Interpretation	
Nausea, retching, vomiting, flatulence	Cyclic vomiting (CVS)	
Seasickness	Vestibular dysfunction (occurs with CVS)	
Headache, visual disturbances	Migrainous (common with CVS)	
Palpitations, pain in the chest	Supraventricular tachycardia (WPW)	
Muscle twitching	Fasciculation	
Lethargy, weakness	Chronic fatigue (CFS)	
Rheumatism, pain in the back	Fibromyalgia (occurs with CFS)	
‘Hezy’, ‘heeziness’, ‘heavy chest’	Bronchospasm	
Eczema of face, lips, hands	Atopic dermatitis	
Anxiety, episodes of fear	Panic attack (DSM-5)	
‘Pins, needles’ hands (paresthesia)	Peripheral neuropathy	
Dizziness, ‘lightheaded’	Hypotension (dysautonomia)	
Shivering, sweating	Dysautonomia	
Transient memory loss, partial paralysis	‘Stroke-like’ episodes	
Sudden facial swelling, redness	Idiopathic flushing	
Abdominal pain – severe, agonizing	Functional abdominal pain	
Secondary symptoms (complications)	Interpretation	
		Vomiting clots blood	Oesophageal tear (Mallory-Weiss)		
Recurrent boils	Complication atopic dermatitis		
Corroded teeth	Dental erosion – regurgitation gastric acid		
Skin pigmentation (‘ruddy’)	Addisonian pigmentation (ACTH/MSH)		
Hysterical sobbing (dacrystic seizures)	Stroke-like episode – temporal lobe		

His most distressing symptoms were gastrointestinal, nausea, retching, vomiting, much flatulence, and at times agonising abdominal pain [4]. Darwin’s illness had many of the characteristics of the cyclic vomiting syndrome (CVS) [8]. Patients with CVS have attacks brought on by stress, even by pleasurable events (‘positive stress’), they suffer from motion sickness, headaches, and some also have rashes and facial swelling, as experienced by Darwin.

At separate times Darwin suffered from spasms of severe abdominal pain. Edward Wickstead Lane (1823-1889), a physician from whom Darwin frequently sought treatment, described this as follows: ‘*In the course of a long professional experience I have seen many cases of violent indigestion, in its many forms, and with the multiform tortures it entails, but I cannot recall any where the pain was so truly poignant as in his. When the worst attacks were on he seemed almost crushed with agony, the nervous system being severely shaken and the temporary depression resulting distressingly great*’ [9].

Diagnoses for Darwin’s Illness

Diagnoses for Darwin’s illness are as numerous and as variable as were his symptoms [10]. Some diagnoses accurately reflect particular symptoms but not the whole extent of the illness [7,11-13].

The diagnosis favoured here is that of adult-onset mitochondrial disorder due to a maternally inherited pathological mitochondrial DNA (mtDNA) mutation, particularly MELAS (mitochondrial encephalopathy, lactic acidosis, and stroke-like episodes) syndrome [5]. This diagnosis accounts for Darwin’s primary symptoms (Table 1) including his cyclic vomiting [14]. Family study shows that this mutation was inherited from his Wedgwood ancestors through his mother Susannah (1765-1817), shared with her siblings Tom Wedgwood (1771-1805) and Mary Ann (1778-1786) [15]. Mary Ann, the youngest sibling, died at the age of eight with typical symptoms of MELAS syndrome [16]. The mutation also manifested in Darwin’s siblings, in particular his elder brother Erasmus Darwin (1804-1881) [5].

Mitochondria and mitochondrial disorders

Mitochondria produce energy for the cell in the form of ATP. Cells vary in the numbers of mitochondria they contain, from nil (erythrocytes) to several thousand, depending on the cell’s energy requirements. Unlike other cell organelles, mitochondria contain their own DNA, mtDNA. MtDNA mutates more commonly than nuclear DNA (nDNA); cells frequently contain both ‘wild’ (normally functioning) DNA and mutant mtDNA, a condition referred to as heteroplasmy [17]. When a cell divides, mitochondria flow randomly to daughter cells so that these may vary in their levels of heteroplasmy.

Mitochondria are maternally derived in humans; the few mitochondria present in the sperm do not survive in the fertilized ovum. Mature ova contain thousands of mitochondria. Due to a reduction rather than a proliferation during maturation, ova from the same mother, even from the same ovary, vary considerably in heteroplasmy levels.

The level of heteroplasmy and the distribution of the mutant DNA in the tissues of the body are partially responsible for similar manifestations of different mutations and, conversely, the same mutation may result in very different symptoms.

Because of variation in heteroplasmy levels in the original ovum, pregnancies in the same mother may result in spontaneous abortion, early fatal childhood disease, progeny developing disease in adult life, or in apparently healthy individuals. Further variation in clinical symptoms occurs as a result of the random distribution of wild and mutant mitochondria in dividing cells.

This variation, both in the severity of disease and the nature of symptoms, is seen with Charles Darwin, his siblings, and his maternal (Wedgwood) forebears [5]. Among these were Charles’ elder brother, Erasmus, who suffered from lethargy and abdominal pains, and their mother, Susannah (1765-1817), who famously was ‘*never quite well & never very ill*’ [18].

MELAS syndrome was one of the first mitochondrial disorders to be recognised [16]. Pathological variants in 17 mtDNA located genes have been identified with the syndrome [19]. As Mary Ann had typical MELAS symptoms, the pathological mutant is assumed to have occurred in one of these 17 known genes, and, with different manifestations, affecting the maternal line family descendants.

Neuroendocrine cells have particularly high energy requirements in the form of ATP. ATP is required not only for cell metabolism and hormone production but also for granule buffering and stability; dense core granules are rich in ATP [20]. If ATP production is diminished, the neuroendocrine function would be compromised. Diabetes is a common manifestation of mitochondrial disorders, occurring as a result of diminished insulin production by the neuroendocrine pancreatic β-cells [21].

## Review

*‘I am a firm believer that without speculation there is no good & original observation’ -* Charles Darwin; letter to Alfred Russel Wallace, December 1857.

General symptoms

Many of Darwin’s symptoms may be traced directly to dysfunction of cells with high energy requirements, including his palpitations to the Purkinje cells responsible for cardiac impulse conduction, headaches and visual disturbances to cerebral endothelial cells responsible for maintaining the blood-brain barrier, and heat and cold intolerance to dysfunction of autonomic neurones. Other symptoms, such as his episodes of profound lethargy and his atopic dermatitis, are not yet explained concerning impaired individual cell or tissue function but are common in patients with mitochondrial disorders [22]. In addition, there are some symptoms that may relate to neuroendocrine cell dysfunction.

Idiopathic flushing

The morning flushing experienced by Darwin appears characteristic of IF, which to an observer may be indistinguishable from flushing associated with a neuroendocrine (carcinoid) tumour (NET). As Darwin’s flushing occurred during mental stimulation, ‘animated discussion’, if the neuroendocrine theory is accepted, the flushing would appear to be an immediate result of excessive vasoactive hormone release.

Aldrich et al. recorded IF in 11 patients in a paper published in 1988 [23]; none of these patients was found to have a carcinoid tumour or any other known cause of flushing. Seven of these patients had additional symptoms suggestive of a panic disorder, including palpitations, five had hypotension with syncope, two had headaches, two had abnormal fatigue and one suffered lightheadedness, all symptoms experienced by Darwin. Biochemical studies showed elevation of serotonin (5-HT) in one patient, vasoactive intestinal peptide in one, substance P (SP) in two, and variable elevations of prostaglandin E and motilin in one. Six patients were treated with SMS 201-995 (Sandostatin-octreotide acetate, Novartis Pharmaceuticals), a potent somatostatin analogue, with the elimination of symptoms or improvement in three patients. The two patients with SP elevation had a return to normal levels, although one showed no change in symptoms. The elevation of some neuroendocrine hormones and response in a proportion of those treated with a somatostatin analogue support the contention that IF has, in at least some cases, a non-NET, neuroendocrine origin.

Patients today, diagnosed as having cyclic vomiting, may experience similar flushing. Patient: ‘I have had unexplained rashes and I get extreme sudden swelling of my face, especially of my lips. … The allergist said it was most likely autoimmune and due to stress’. The patient’s mother: ‘His face looks like he got hit by Rocky Balboa ... his lips are red, bleeding and swollen’ (‘Rocky Balboa’ is a fictional southpaw boxer in a film series) [24]. Darwin was not alone in experiencing IF as an added suffering to his cyclic vomiting.

Hormones contained in neurosecretory granules are thought to be released by piecemeal degranulation [25]. If there is a deficiency of ATP and resulting granule instability, then uncontrolled rather than piecemeal release could occur following stimulation. Patients who experience flushing and facial oedema, as well as cyclic vomiting, may, like Darwin, have a mitochondrial disorder.

The presence of elevated neuroendocrine hormone levels and the response to somatostatin analogues in these patients, although hardly conclusive, support the contention that IF, and Charles Darwin’s unusual but similar symptoms, have neuroendocrine pathogenesis.

Panic attacks

The flushing may not be the only symptom related to neuroendocrine dysfunction. Darwin wrote: ‘*I have awakened in the night being slightly unwell and felt so much afraid though my reason was laughing and told me there was nothing and tried to seize hold of objects to be frightened of*’ [7]. This description is characteristic of a panic attack. Darwin had other symptoms that occur with a panic disorder, with dying sensations, trembling, sweating, and palpitations (DSM-5). Such episodes are known to occur in patients with a mitochondrial disorder [22].

Darwin’s attacks of fear may be related to cholecystokinin (CCK) activity. CCK was one of the first peptide hormones to be identified. It is secreted by neuroendocrine cells in the duodenal and jejunal mucosa; by neurons of the cerebral cortex, hippocampus, hypothalamus, or elsewhere in the brain; and by other cells in various tissues [26]. Along with its intestinal effects of gallbladder contraction and pancreatic secretion, it acts as a neurotransmitter and has anti-inflammatory and renal natriuretic properties. CCK is intimately involved in diverse normal behaviours such as learning and memory, feeding, satiety, and detection of painful stimuli; it is also strongly linked to several central nervous system disorders, including anxiety and panic attacks. It was shown to induce panic when infused into healthy volunteers [27]. Moreover, its intestinal actions are inhibited by somatostatin; cerebral inhibition is less well understood but somatostatin is expressed in inhibitory neurons in the cerebral cortex, hippocampus, hypothalamus, and other areas [28].

The fact that these attacks occurred at night, waking Darwin from sleep, suggest that this symptom may have been due to failure of inhibition rather than excessive or inappropriate release of the hormone.

Gastrointestinal symptoms and abdominal pain

Darwin’s gastrointestinal symptoms, with episodic nausea, retching, vomiting, and flatulence are much the same as those in patients diagnosed today as having CVS [8]. These patients frequently also suffer from motion sickness including seasickness (as did Darwin) and have the unusual feature of attacks of illness being brought on by pleasurable events. Darwin’s gastrointestinal symptoms are proposed as being characteristic of CVS [13], a disorder that has been linked to mitochondrial disorders in several instances [29,30].

Severe, acute abdominal pain, as experienced by Darwin, was described in 1966 in a patient with metastatic NET, occurring independently of recurrent flushing and of any bowel obstruction that may result from a carcinoid tumour [31]. The symptom of severe, agonising abdominal pain, not related to defecation, differs from the pain experienced in irritable bowel syndrome and is more characteristic of functional abdominal pain syndrome (FAPS) [32]. The pain in FAPS is described as ‘agonising’, ‘the worst ever’, and the condition may be associated with fibromyalgia and chronic fatigue, symptoms of which were also experienced by Darwin (Table 1). Abdominal pain is recognised as occurring today in patients with a known mitochondrial disorder [33].

These upper intestinal symptoms may relate, in part at least, to the action of 5-HT. Approximately 90% of the body’s 5-HT is found in neuroendocrine cells of the intestine where it has a complex physiological action [34]. 5-HT is also present in the enteric nervous system as well as the central nervous system. The coordinated movement of food along the gastrointestinal tract depends on 5-HT-mediated regulation of smooth muscle tone, peristalsis, mucosal secretion, and visceral perception via an interaction with intrinsic enteric and extrinsic afferent neurons, the interstitial cells of Cajal, smooth muscle cells, and intestinal epithelial cells [35]. 5-HT has been linked to the pathogenesis of several functional bowel disorders, including irritable bowel syndrome and cyclic vomiting. Patients with CVS improve with therapy including 5-HT receptor antagonists [36].

Darwin’s vomiting occurred several hours after eating, and what he vomited was mostly mucous and bile with little food. He wrote to his friend Hooker in February 1864: *‘it rarely comes on till 2-3 hours after eating, so that I seldom throw up food, only acid & morbid secretion; otherwise I should have been dead, for during a month I vomited after every meal & several times most nights*’ [37]. This timing would be consistent with 5-HT release into the jejunal mucosa, which occurs after mechanical stimulation such as a bolus of food entering the small intestine [35].

The patient described in 1966 with agonising abdominal pain obtained only minimal relief with analgesics but this symptom was completely abated by intravenous methocarbamol (Robaxin), a muscle relaxant [31]. 5HT indirectly stimulates circular smooth muscle, and spasms due to uncontrolled release may produce this symptom. The vomiting, occurring several hours after eating, may have a similar hormonal mechanism and respond to the same therapy.

## Conclusions

The numerous symptoms experienced by Charles Darwin in the course of his chronic relapsing illness may be related to a mitochondrial disorder with resultant dysfunction of cells or tissues that have high energy requirements.

Darwin’s facial flushing, his panic attacks, his episodes of agonising abdominal pain, and his upper intestinal symptoms may be due to neuroendocrine hormone imbalance, either uncontrolled hormone release or failure of inhibition.

Patients today with similar diverse symptoms should be investigated for a mitochondrial disorder and may benefit from therapy with somatostatin analogues or 5-HT receptor antagonists. Elevated vasoactive or peptide hormone levels and response to therapy would support the contention that these distressing complaints have a neuroendocrine basis.
